# SPR741, Double- or Triple-Combined With Erythromycin and Clarithromycin, Combats Drug-Resistant *Klebsiella pneumoniae*, Its Biofilms, and Persister Cells

**DOI:** 10.3389/fcimb.2022.858606

**Published:** 2022-03-18

**Authors:** Pengfei She, Yaqian Liu, Lanlan Xu, Yimin Li, Zehao Li, Shasha Liu, Zubair Hussain, Yong Wu

**Affiliations:** ^1^ Department of Laboratory Medicine, The Third Xiangya Hospital of Central South University, Changsha, China; ^2^ Department of Laboratory Medicine, The First Hospital of Changsha, Changsha, China

**Keywords:** *Klebsiella pneumoniae*, SPR741, macrolide, triple antibiotic combination, extensively drug-resistant, pandrug-resistant, biofilm, persisters

## Abstract

*Klebsiella pneumoniae* has emerged as a major clinical and public health threat owing to the increasing prevalence of healthcare-associated infections caused by multidrug-resistant or extensively drug-resistant strains. However, increasing antibiotic resistance and the absence of clinically effective antimicrobial agents make combination therapy an urgent need. This study investigated the anti-microbial activity of SPR741, a polymyxin B derivative, in combination with macrolide antibiotics (erythromycin and clarithromycin), against extensively drug-resistant and pandrug-resistant *K. pneumoniae.* Monotherapy, double, and triple combination therapies were performed to identify the most effective treatment combination using *in vitro* checkerboard, time-killing kinetics. Furthermore, we evaluated the biofilm eradication and persister cell-killing activity of these combinations using laser confocal microscopy and colony forming unit counting. In addition, a neutropenic mouse thigh infection model was used to assess the therapeutic efficacy and toxicity of the triple antibiotic combination against pandrug-resistant *K. pneumoniae in vivo*. Our results suggested that SPR741 combined with macrolides exhibited strong synergistic antibacterial activity against extensively drug-resistant and pandrug-resistant *K. pneumoniae*. These antibiotic combinations could also effectively eradicate highly resistant bacterial biofilms and persister cells *in vitro* and demonstrate considerable efficacy and low toxicity *in vivo*. In summary, our findings indicated that SPR741, in combination with macrolide antibiotics (double or triple combination), has the potential to serve as a novel treatment option against drug-resistant *K. pneumoniae* -related infections.

## Introduction

*Klebsiella pneumoniae* is an opportunistic pathogen and a major cause of hospital-acquired infections (Arato et al., 2021). *K. pneumoniae* has long been recognized as a causative agent of pneumonia and causes bacteremia, urinary tract infections, and neonatal sepsis (Paczosa and Mecsas, 2016). Over the past few decades, extensively drug-resistant (XDR) and pandrug-resistant (PDR) *K. pneumoniae* have become a notable burden on healthcare worldwide (Koskinen et al., 2021). The World Health Organization has listed carbapenemase-producing *K. pneumoniae* as a critical public health threat (World Health Organization, 2017); infections caused by *K. pneumoniae* strains that have developed resistance against new generations of β-lactams, such as carbapenems, can be hazardous and often life-threatening. Although colistin has achieved remarkable clinical success (Falagas et al., 2010), development of resistance against it has been reported (Longo et al., 2019; Hamel et al., 2020), attributed to its widespread nosocomial application. This looming health threat has demanded the development of new anti-microbial therapies to treat intractable *K. pneumoniae -*related infections.

The management of infections caused by *K. pneumoniae* is highly challenging, as this pathogen can form biofilms, and thus, colonize tissues and medical devices (Guilhen et al., 2019). Biofilms are microcolonies in which cells are embedded in an extracellular polymer matrix and constitute a significant resistant phenotype (Geladari et al., 2019); these are 100 to 1000 times more tolerant to conventional antibiotics than their planktonic counterparts (Olsen, 2015). In addition, the bacteria inside the biofilm easily form persister cells, a phenotypical variant subpopulation of bacteria in a dormant and antibiotic-tolerant state that largely contributes to the recalcitrance and relapse of biofilm-associated infections, which are difficult to eradicate with the currently available anti-bacterial agents (de Breij et al., 2018; Soares et al., 2020). Thus, there is an urgent need for novel strategies that specifically target biofilms and persister cells.

SPR741 is a novel polymyxin B (PB) derivative that exhibits minimal intrinsic Gram-negative anti-bacterial activity, but interacts with the outer membrane and damages the integrity of lipopolysaccharide, thereby permeabilizing the outer membrane and enabling the entry of anti-bacterial agents (Vaara, 2013; Mendes et al., 2020a). SPR741 retains the cyclic peptide portion of PB but it has a reduced positive charge and lacks the highly lipophilic fatty acid side chain, thus reducing its nephrotoxicity (Zurawski et al., 2017). Although, activity of SPR741, in combination with conventional antibiotics, has been reported against Gram-negative pathogens (Stainton et al., 2018; Mendes et al., 2020b). The effects of a triple antibiotic combination with SPR741 have not yet been reported. In addition, the effects of the antibiotics combined with SPR741 against biofilm and persister cells have also not been reported.

Owing to their high efficiency and safety, macrolide antibiotics have been widely used in clinical practice for over 50 years. These belong to the largest category of antibiotics and are particularly suitable for the treatment of upper and lower respiratory tract infections (Jednačak et al., 2020). For example, azithromycin (AZM) has been shown to prevent *Pseudomonas aeruginosa* ventilator-associated pneumonia by inhibition of quorum sensing (QS) (van Delden et al., 2012). Furthermore, it has been reported that macrolide antibiotics used at sub-MIC concentrations can inhibit *P. aeruginosa* virulence and biofilm formation (Nalca et al., 2006; Baldelli et al., 2020). Thus, we hypothesized that the combination of SPR741 and erythromycin (E) and clarithromycin (CLA) might also have the potential to combat biofilms.

Previous studies have reported the superior activity of antibiotic combinations for the treatment of bacterial infections. Drug combinations may directly or indirectly block the mechanism of antibiotic resistance and prevent resistance (Vipin et al., 2020). Moreover, drug combination could significantly reduce the required dose, improve patient compliance, and thereby, reduce side effects and cytotoxicity (She et al., 2019). Several synergistic combinations have been successfully used for PDR Gram-negative pathogen infections, such as double carbapenem or double carbapenem with colistin, to treat carbapenemase -producing *K. pneumoniae*. Synergistic combinations are often the only treatment option for PDR Gram-negative infections (Karakonstantis et al., 2020). Evidently, drug combination has a broad prospect for clinical application, to combat XDR or PDR bacterial infections.

In this study, we investigated the anti-bacterial activity of SPR741, in combination with macrolide antibiotics (double or triple combination), against XDR and PDR *K. pneumoniae*, its biofilms, and persister cells. To the best of our knowledge, the effects of a triple antibiotic combination with SPR741 against biofilm and persister cells of *K. pneumoniae* have not been reported yet. In this study, we found that SPR741 exhibited the most significant anti-bacterial activity when combined with two macrolides (E and CLA), and could synergistically eradicate mature biofilms and kill persister cells. SPR741 combined with E and CLA also showed a significant synergistic anti-microbial effect *in vivo*, in a neutropenic murine thigh infection model of PDR *K. pneumoniae*.

## Materials and Methods

### Bacterial Strains and Culture Conditions

The type strain of *K. pneumoniae* ATCC 4352 was purchased from American Type Culture Collection. *K. pneumoniae* ATCC 700603 was provided by Juncai Luo (Tiandiren Biotech, Changsha, China). *K. pneumoniae* XDR clinical isolate strains of KPLUO and KPWANG were provided by Cha Chen (Traditional Chinese Medicine Hospital of Guangdong Province, Guangzhou, China). *K. pneumoniae* PDR clinical strain LH2020 was collected from the Third Xiangya Hospital of Central South University (Changsha, China). The *K. pneumoniae* clinical isolates of KPLUO, KPWANG and LH2020 were both obtained from the sputum of hospitalized patients with lung infections from Jan to Apr in 2020. K*. pneumoniae* strains were cultured in Luria-Bertani (LB) broth (Solarbio, Shanghai, China). All bacteria were grown at 37°C with shaking at 180–200 rpm. SPR741, E, CLA, and other drugs (including PE: Polymyxin E; PMBN: Polymyxin B nonapeptide) were purchased from MedChem Express (NJ, USA).

### Antibiotic Susceptibility Test

The minimal inhibitory concentrations (MICs) of antibiotics were determined using broth microdilution assay (Song et al., 2020), as recommended by the Clinical and Laboratory Standards Institute (CLSI, 2021). Briefly, bacteria were cultured to mid-log phase in LB medium at 37°C, 180 rpm, and then diluted to approximately 1×10^6^ CFU/mL. The tested antibiotics were serially diluted 2-fold in Mueller-Hinton (MH) broth II (cation regulation) and mixed with an equal volume of bacterial suspension in a 96-well plate (Corning Costar, USA). The MIC was defined as the lowest concentration without visible bacterial growth after incubation at 37°C for 16–18 h. To determine the minimum bactericidal concentration (MBC), 10 μL of bacterial culture was spotted on sheep blood agar plates (AutoBio, Zhengzhou, Henan, China). MBC is considered the minimum drug concentration without bacterial colony growth on the plate, after incubation at 37°C overnight.

### Kirby–Bauer (K-B) Disk Diffusion Test

An overnight culture of *K. pneumoniae* was adjusted to a McFarland standard equivalent of 0.5, and then spread onto a MH agar plate using a sterile cotton swab. The blank disks loaded with antibiotics were manually placed on the surface of agar and incubated at 37°C for 16-18 h. Then, the outcomes were determined according to diameter of inhibition zones (CLSI, 2021).

### Checkerboard Assay for Double and Triple Antibiotic Combination

A microdilution checkerboard assay was used to assess the interactions of double or triple antibiotic combinations against *K. pneumoniae* isolates. For the double antibiotic combination, bacterial suspension at mid-log phase was diluted to 1×10^6^ CFU/mL with MH broth, 50 μL of 2-fold serially diluted drug added horizontally to the 96-well plate, and another 50 μL serially diluted drug with bacterial suspension was added vertically to the well. After incubation at 37°C for 16–18 h, the results were determined by measuring the optical density at 630 nm (OD_630_) using a microplate spectrophotometer (Bio-Rad, USA), and the fractional inhibitory concentration index (FICI) was calculated using the formula: FICI = MIC_A_ (in combination)/MIC_A_ (singly) + MIC_B_ (in combination)/MIC_B_ (singly). FICI was judged as follows: FICI ≤ 0.5, synergism; 0.5<FICI ≤ 1, additive; 1<FICI ≤ 4, indifference; and >4, antagonism (Dupieux et al., 2017). For the triple antibiotic combination (SPR741+E+CLA), antibiotic E was added to the MH medium at the expected concentration, and the other two diluted drugs (SPR741 and CLA) were added separately to the 96-well plate, as described previously for the double antibiotic combination. OD_630_ was detected after incubation at 37°C for 16–18 h.

### Time-Killing Assay

A single colony of *K. pneumoniae* was inoculated in 10 mL LB medium and cultured overnight at 37°C, with shaking at 180 rpm. The bacterial cells were diluted to 1×10^6^ CFU/mL, and mono- and combinational therapy of SPR741, CLA, and E were added to 5 mL of suspension; 0.1% dimethyl sulfoxide (DMSO) was added as a control treatment. Samples were collected at 0, 2, 4, 6, 8, and 24 h, and viable counts were determined by means of plate counting (Liu et al., 2020).

### Biofilm Eradication Assay

The biofilm eradication assay was performed as described previously (Chatzimoschou et al., 2015), with minor modifications. Specifically, *K. pneumoniae* cultured overnight was diluted 1:100 with MH broth containing 2% glucose, following which 100 µL bacterial suspension was added to the 96-well microplates and incubated at 37°C for 48 h, to form mature biofilms. The unattached planktonic cells were removed by washing with 1× PBS (pH=7.4) twice, following which 100 µL of MH broth containing antimicrobials was added at the indicated concentrations. After incubation at 37°C for 24 h, the unattached cells were gently washed with 1× PBS, and the biofilm biomass was determined by measuring the OD_630_.

### Determination of Viable Cells in Biofilms

The 48-h pre-formed biofilms were established as previously described. After treatment with the indicated concentrations of antibiotics alone or in combination for 24 h, the biofilms were washed with 1×PBS and dispersed by vigorous pipetting in 100 µL saline, to ensure separation from the wall of each well. The bacterial mixtures were subsequently transferred to a new 96-well microplate, continuously diluted 10-fold in saline, following which the viable bacterial cells were counted by means of plate counting (Tan et al., 2019).

### Confocal Laser Scanning Microscopy (CLSM)

CLSM was used to visually evaluate the effect of SPR741 combined with macrolides in eradicating pre-formed biofilms. The overnight culture of *K. pneumoniae* was diluted 1:100 with MH broth containing 2% glucose, following which 2 mL of *K. pneumoniae* cultures were subsequently added to a 6-well microplate (Corning Costar, USA), and the sterile glass sheet was placed into the wells. After incubation at 37°C for 48 h, the bacterial suspension was subjected to mono-, dual- and triple-therapy with SPR741, CLE, and E; 1% DMSO treatment served as a control. After incubation for 24 h, a mixture of SYTO9 and Propidium Iodide (PI) (Thermo Fisher Scientific, Shanghai, China) was used to dye the biofilm for 15 min in the dark. The biofilms were visualized using CLSM (Zeiss LSM800, Jena, Germany), with excitation and emission wavelengths of 485 nm/530 nm and 485/630 nm for SYTO9 and PI, respectively. (Liu et al., 2015).

### Persister Cell-Killing Assay

Carbonyl cyanide 3-chlorophenylhydrazone (CCCP)-induced persister cells were cultured as previously described (Narayanaswamy et al., 2018), and overnight cultures of *K. pneumoniae* were incubated with 200 μg/mL CCCP at 37°C, with shaking at 180 rpm for 3 h, to induce persister cell formation. The bacteria were then centrifuged, washed twice with saline, and re-suspended in MH medium to obtain a final density of 1×10^8^ CFU/mL. Drugs were added at the indicated concentrations and the cells were incubated with them for 6 h, following which the surviving persister cells were determined by means of plate counting method.

### Neutropenic Murine Thigh Infection Model

All animal procedures were performed in accordance with the Ethics Committee of the Third Xiangya Hospital, Central South University (no: 2019sydw0233). Specific pathogen-free ICR female mice (Hunan, China), aged 8 weeks and weighing 24–26 g, were used. Mice (n = 6 -7 mice/group) were rendered transiently neutropenic by intraperitoneal injection of cyclophosphamide at a dose of 150 mg/kg and 100 mg/kg, at 4 d and 1 d before bacterial infection. To induce deep thigh infection, overnight culture of *K. pneumoniae* LH2020 was adjusted to a concentration of approximately 1×10^6^ CFU/mL, and the final inoculum concentrations were confirmed by means of serial dilution and plating techniques. Fifty microliters of the bacterial suspension was then injected intramuscularly into the right thigh muscle of the mice. Anti-microbial therapy was initiated 1 h after the infection. Treatments with 30 mg/kg SPR741, 40 mg/kg CLA, and 30 mg/kg E or their combinations were administered by means of subcutaneous injection into the neck (Wang et al., 2019). Saline with 1% DMSO was used as the negative control. Mice were euthanized after 24 h, and the thigh muscles were excised and homogenized with saline. The number of viable cells was counted using the plate counting method.

### *In Vivo* Toxicology Assay

ICR mice weighing 24–26 g were randomly divided into two groups (n = 5 mice/group): the test group was administered a subcutaneous injection of 30 mg/kg SPR741 combined with 40 mg/kg CLA and 30 mg/kg E. Normal saline with 1% DMSO was included as a vehicle control. After 24 h of administration, plasma and serum samples were collected for whole blood cell analysis and alanine aminotransferase (ALT), blood urea nitrogen (BUN), and creatine kinase (CK) levels were measured in them. The mice were euthanized, and the liver, kidney, spleen, heart, and lungs were excised and preserved in 4% paraformaldehyde (Servicebio, Wuhan, China), sectioned, and stained with hematoxylin-eosin for histopathological analysis, and the results were confirmed by a pathologist.

### Statistical Analysis

All experiments were performed at least three independent experiments. GraphPad Prism software 8.0 was used for statistical analysis, and all data were expressed as means ± S.D. Unless otherwise noted, *P*-values were calculated using Student’s *t*-test for two-group comparisons, while multiple comparisons was analysed using one-way ANOVA followed by Tukey tests or Dunn’s multiple comparison test. *P*<0.05 was considered statistically significant.*: *p*<0.05; **: *p*<0.01; ***: *p*<0.001; ****: *p*<0.0001.

## Results

### Synergistic Anti-Microbial Activity of Double and Triple Antibiotic Combinations Among SPR741 and Macrolides Against XDR and PDR *K. pneumoniae*

**Figure 1:**
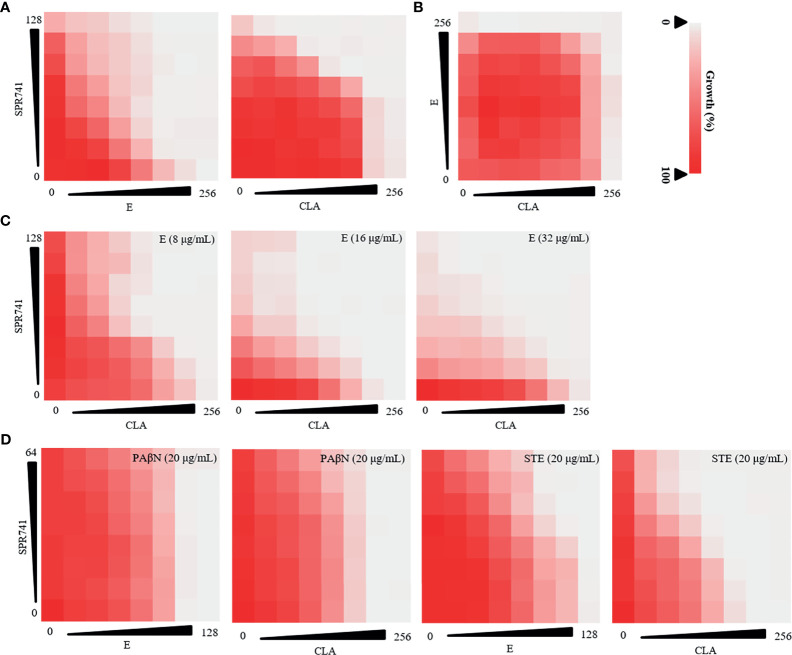
Anti-bacterial effect of double or triple combination against PDR *K. pneumoniae* LH2020 was determined using checkerboard assay. Double antibiotic combination of SPR741 with CLA or E **(A)** and CLA combined with E **(B)** against *K. pneumoniae* LH2020. **(C)** Triple antibiotic combination of SPR741 with CLA and various concentrations of E. **(D)**Triple drug combination based on SPR741, CLA/E combined with 20 µg/mL efflux pump inhibitor PAβN/STE. Experiment was repeated three times.

The antibiotic resistance pattern of *K. pneumoniae* LH2020, KPLUO and KPWANG clinical isolates were confirmed by K-B test and indicated LH2020 was PDR *K. pneumoniae*, KPWANG and KPLUO were XDR *K. pneumoniae* (**Table 1**). We attempted to identify polypeptide antibiotics [including PE, PB, PMBN, and SPR741] that had a synergistic combination with macrolide antibiotics against PDR *K. pneumoniae* LH2020. The results are shown in **Table 2** and **Figure S1**, only SPR741 showed a synergistic combination with macrolide antibiotics. Although SPR741 and macrolide antibiotics lack of intrinsic antibacterial activity against *K. pneumoniae*, SPR741 could sensitized macrolide antibiotics to *K. pneumoniae*, and showed effective synergistic anti-microbial effects with E or CLA (**Figure 1A**), while other polypeptide antibiotics such as PE, PB, and PMBN only showed additive or indifferent effects (**Figure S1**). Therefore, we chose SPR741 as a candidate antibiotic combination. Interestingly, E combined with CLA did not exhibit synergistic activity (**Figure 1B**), whereas the triple antibiotic combination of SPR741 with CLA and E showed the most potent synergistic effect (**Figure 1C**). To exclude the effect of the solvent DMSO, we added DMSO in checkerboard assay and found that DMSO did not affect the OD_630_ value (*P*>0.05), indicating that the presence of DMSO did not affect the results of the combination **(** **Figure S2**). To better compare the synergetic antibacterial difference between the dual- and triple-combinations, we set the MIC value >128 µg/mL for SPR741 in the checkerboard assay to equal 128 µg/mL to calculate the FIC values. As shown in **Table S1**, when 8, 16 and 32 µg/mL of E were added, the FIC values for the triple antibiotic combinations were 0.187, 0.125 and 0.125, respectively, which was lower than that of the dual-combinations, with FIC values of 0.375 (SPR741+CLA) and 0.266 (SPR741+E), indicating that the triple-drug combination may have better antibacterial efficacy than the dual-combinations.

**Table 1:** Resistant pattern of XDR/PDR *K. pneumoniae* clinical isolates.

Strains	AK	AMP	ATM	C	CIP	CRO	CZA	FOS	IPM	LEV	MH	PB	SAM	SCF	SXT	TGC	TZP
LH2020	R	R	R	R	R	R	I	R	R	R	I	R	R	R	R	R	R
KPLUO	R	R	R	R	R	R	S	R	R	R	I	S	R	R	R	I	R
KPWANG	R	R	R	R	R	R	S	R	R	R	I	S	R	R	R	I	R

AK, Amikacin; AMP, Ampicillin; ATM, Aztreonam; C, chloramphenlcol; CIP, Ciprofloxacin; CRO, Ceftriaxone; CZA, Ceftizoxime; FOS, Fosfomycin; IPM, Imipenem; LEV, Levofloxacin; MH, Minocycline; PB, Ploymyxin B; SAM, Ampicillin-Sulbactam; SCF, Cefpoerazone-Sulbactam; SXT, Trimethoprim-Sulfamethoxazole; TGC, Tigecycline; TZP, Piperacillin-Tazobactam; R, resistance; I, intermediate; S, sensitive.

**Table 2:** Antibiotics combination against PDR *K. pneumoniae* LH2020.

Drugs	MIC_singly_	MIC_in combination_	MIC_singly_/MIC_incombination_	FICI	Outcome
PE E	256 128	256 128	1 1	2	Indifference
PE CLA	256 256	64 128	0.25 0.5	0.75	Additive
PMBN E	>256 256	>256 256	1 1	2	Indifference
PMBN CLA	>256 128	>256 128	1 1	2	Indifference
PB E	256 128	128 64	0.5 0.5	1	Additive
PB CLA	256 256	32 128	0.125 0.5	0.625	Additive
SPR741 E	>128 256	2 64	<0.0156 0.25	<0.266	Synergy
SPR741 CLA	>128 256	32 32	<0.25 0.125	<0.375	Synergy

PE, Polymyxin E; CLA, Clarithromycin; E, Erythromycin; PMBN, Polymyxin B nonapeptide; PB, Polymyxin B.

As efflux pump inhibitors have been reported to reduce antibiotic resistance (Lowrence et al., 2019), a checkerboard dilution assay was also conducted to study the triple anti-microbial combinational effects of SPR741+ macrolide (E or CLA) + efflux pump inhibitors (PAβN/STE) against PDR *K. pneumoniae* LH2020, to identify the most active combination *in vitro*. We found that although the double drug combination of SPR741 with E/CLA had a synergistic effect, the combined anti-microbial effect was not enhanced when PAβN or STE was added (**Figure 1D**), besides, synergistic effect of SPR741+C or SPR741+E diminished after the addition of PAβN, suggesting that there may be an antagonistic effect. Hence, in the following study, we selected the anti-microbial agents SPR741, E, and CLA, to investigate their double or triple synergistic anti-bacterial effect.

**Figure 2:**
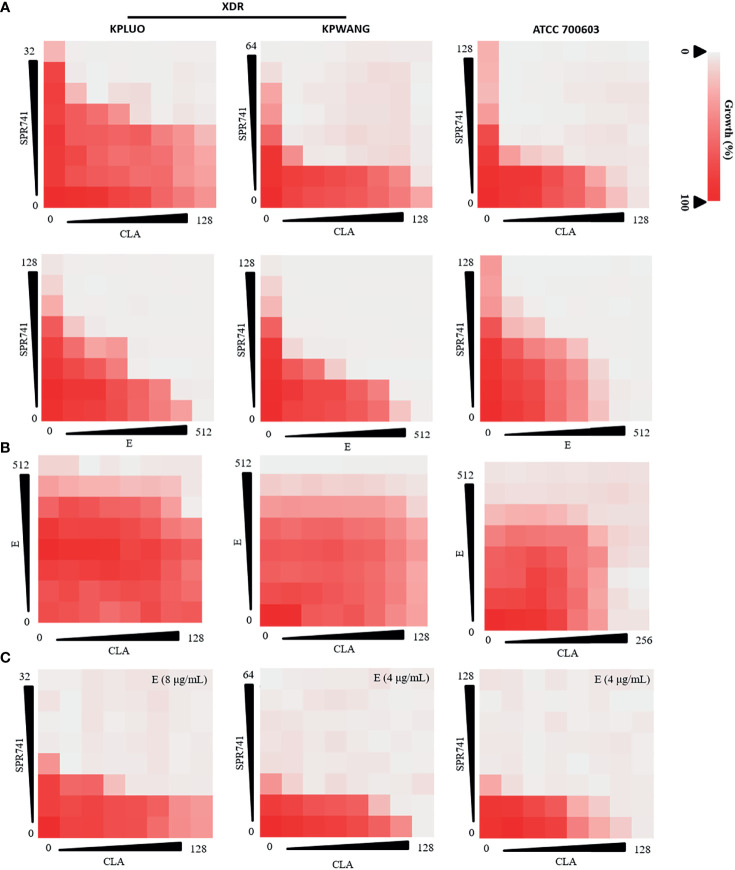
Combination anti-microbial activities of SPR741, CLA, and E against XDR and type strain of *K. pneumoniae*. Double antibiotic combination of SPR741 with CLA or E **(A)**, combination of CLA and E **(B)**, and triple combination of SPR741 with CLA and different concentrations of E **(C)** against XDR *K. pneumoniae* KPLUO, KPWANG, and type strain ATCC 700603. Experiment was repeated three times.

SPR741 also showed a synergistic anti-bacterial effect when combined with E or CLA against XDR *K. pneumoniae* KPLUO, KPWANG, and type strain ATCC 700603 (**Figure 2A**). Although the combination of E and CLA showed no interaction (**Figure 2B**), the anti-bacterial activity was significantly improved by the triple antibiotic combination of SPR741, E, and CLA (**Figure 2C**). Compared with any pair of double antibiotic combinations, the triple antibiotic combination exhibited the most significant anti-microbial effect. As seen in **Table S2**, for the clinical isolate KPLUO, the FICI values for SPR741 in combination with CLA or E were 0.187 and 0.156, respectively, and when 8 µg/mL E was added, a lower FIC value of 0.0468 was observed. Similarly, the FIC values for the triple-antibiotic combination groups were lower than that of the dual-antibiotic combination for *K. pneumoniae* KPWANG and ATCC 700603, demonstrating that E enhanced the antibacterial activity of the SPR741 + CLA, which indicates the synergistic antimicrobial activity of the triple-antibiotic combination. Besides, we have selected four multi-drug resistant clinical strains of *K. pneumoniae* to determine whether the synergistic antibacterial effects were strain-dependent (**Figure S3**), interestingly, synergistic antimicrobial activity was still observed with triple combination therapy, it demonstrates triple combinations may achieve better antimicrobial efficacy than double-drug combinations.

### Synergistic Killing Activity of Triple Antibiotic Combination Among SPR741, E, and CLA

**Figure 3:**
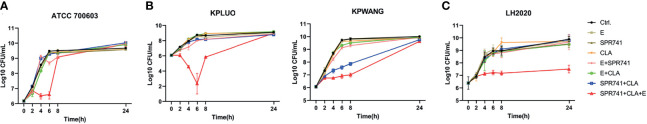
Triple antibiotic combination exhibits bactericidal activity against XDR and PDR *K. pneumoniae.* Time-killing curve of monotherapy, double, and triple combination therapies based on 7 µg/mL SPR741, 5 µg/mL CLA, and 7 µg/mL E against type strain of ATCC700603 **(A)**, and 2 µg/mL SPR741, 2 µg/mL CLA, and 4 µg/mL E against XDR *K. pneumoniae* KPLUO and KPWANG **(B)**; PDR *K. pneumoniae* LH2020 was treated with 8 µg/mL SPR741, 8 µg/mL CLA, and 16 µg/mL E; bacterial suspension was treated with 0.1% DMSO served as a control **(C)**. Samples were harvested, diluted, and counted at time-points of 0, 2, 4, 6, 8, and 24 h. Experiment was repeated three times.

To verify the combined effects of SPR741 with E and CLA, we performed a time-killing assay. The results showed that the triple antibiotic combination group showed the most effective bactericidal activity against XDR and PDR *K. pneumoniae* compared with mono- or double- antimicrobials treated groups. For the type strain ATCC 700603, the triple combination of SPR741 (7 µg/mL), CLA (5 µg/mL) and E (7 µg/mL) reduced the bacterial count by 2.88 Log10 CFU/mL at 6 h, compared to the control group (**Figure 3A**). For XDR strains KPLUO and KPWANG, the single or double combination of SPR741 (2 µg/mL), CLA (2 µg/mL) and E (4 µg/mL) showed no bactericidal activity, but the triple drug group reduced the number of bacteria from over 8 to 2.34 Log10 CFU/mL (KPLUO), and over 9 to 6.91 Log10 CFU/mL (KPWANG), respectively, after treatment for 6 h (**Figure 3B**). Similarly, for the PDR strain LH2020, the triple combination groups among SPR741 (8 µg/mL), CLA (8 µg/mL) and E (16 µg/mL) could also reduce the number of bacteria from over 8.81 to 7.18 Log10 CFU/mL at the time point of 6h (**Figure 3C**).

### Persister Cell-Killing Activity of the Double or Triple Combination of SPR741 and CLA/E

**Figure 4:**
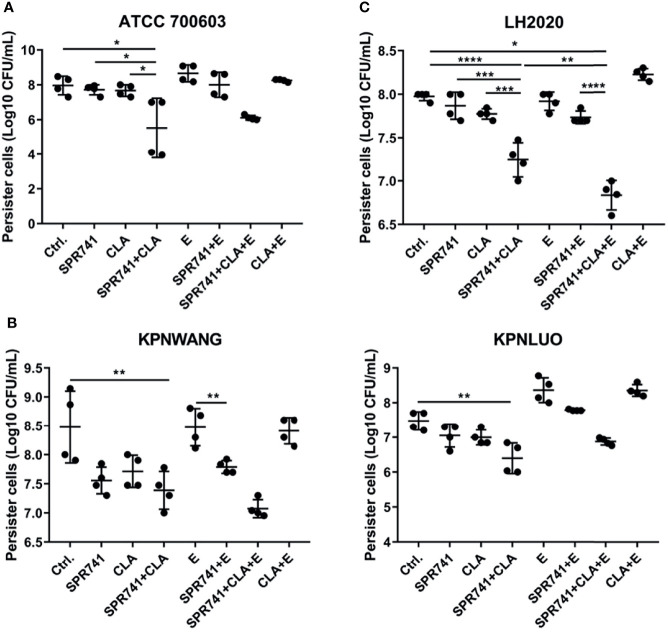
SPR741 combined with CLA and E shows strong bactericidal activity against CCCP-induced *K. pneumoniae* persister cells. Viability of *K. pneumoniae* ATCC 700603 persister cells exposed to 8 µg/mL SPR741, 16 µg/mL CLA, and 16 µg/mL E for 6 h; the control group was left untreated **(A)**; The killing effect of 16 µg/mL SPR741, 32 µg/mL CLA, and 32 µg/mL E on XDR *K. pneumoniae* KPWANG and KPLUO **(B)** and PDR *K. pneumoniae* LH2020 **(C)** persister cells. Data are presented as mean ± S.D and analysed by Dunn’s multiple comparison test. Experiment was repeated four times. *p < 0.05; **p < 0.01; ***p < 0.001; ****p < 0.0001.

Based on the significant synergistic effect of the triple combination on the planktonic bacteria of *K. pneumoniae*, we proceeded to ask whether this combination was effective against highly resistant phenotype of persister cells. We therefore investigated the synergistic bactericidal activity against CCCP-induced persisters by means of colony counting. The persister cell-killing assay showed that, in contrast to the control or single used group, the double or triple combination based on SPR741, CLA and E showed the greatest bactericidal effect against persister cells of type strain ATCC 700603 (**Figure 4A**), XDR (**Figure 4B**), and PDR (**Figure 4C**) strains. For example, in case of the ATCC 700603 (**Figure 4A**), whether it was the control or single drug group, the number of persister cells was between 7.6 to 8.6 Log10 CFU/mL, while the combination of 8 µg/mL SPR741 and 16 µg/mL CLA could significantly reduce the number of persisters to 5.5 Log10 CFU/mL. In case of the XDR strain of *K. pneumoniae* KPWANG and KPLUO, as compared to the control, the combination of 16 µg/mL SPR741 and 32 µg/mL CLA could reduce the number of persisters by 1.1 and 1.08 Log10 CFU/mL, respectively (**Figure 4B**), while the addition of 32 µg/mL E reduced the persisters by 1.41 and 0.58 Log10 CFU/mL. As for the PDR strain *K. pneumoniae* LH2020, the combination of 16 µg/mL SPR741 and 32 µg/mL CLA could reduce the number of persisters by 0.74 Log10 CFU/mL, when 32 µg/mL of E was added, the bactericidal potency against persister cells was further enhanced, as confirmed by reduced the bacterial count by 1.14 Log10 CFU/mL (**Figure 4C**). Although the triple combination did not significantly enhance the persisters-killing activity against the type strain ATCC 700603 and the XDR strain KPLUO, the double combination of SPR741 and CLA was effective enough in killing persister cells.

### Effective Anti-Biofilm Activity of SPR741 Combined With CLA and E

**Figure 5:**
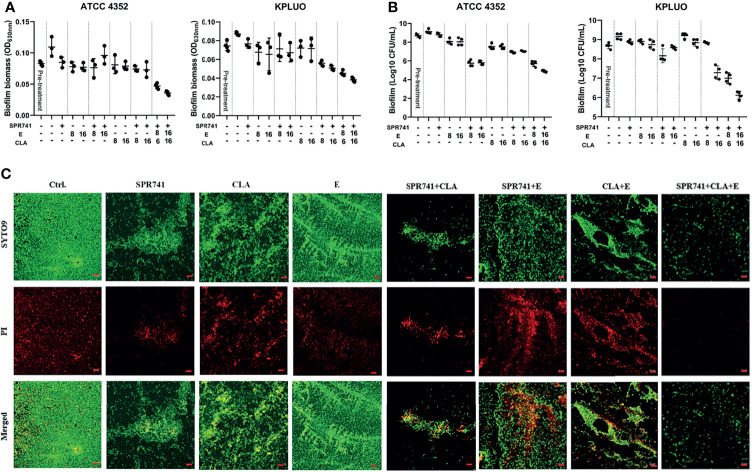
Synergistic biofilm eradication and killing activity of SPR741, in combination with CLA and E, against *K. pneumoniae*. Effects of 8 µg/mL SPR741 combined with CLA (8/16 µg/mL) or E (8/16 µg/mL), in terms of eradicating 48 h pre-formed biofilm **(A)** and cell-killing **(B)**, in *K. pneumoniae* ATCC 4352 and XDR strain KPLUO. Experiment was repeated three times. **(C)** Representative CLSM images of anti-biofilm effects of SPR741+CLA+E-based mono, double or triple combination against ATCC 4352. Scale bar: 20 µm.

*K. pneumoniae* ATCC 4352 and KPLUO were chosen for biofilm eradication and viable bacteria counting assays due to their strong biofilm-forming ability, while *K. pneumoniae* KPWANG, LH2020 and ATCC 700603 had a weaker biofilm forming ability. When 8 µg/mL SPR741 was combined with 16 µg/mL CLA and 16 μg/mL E, the combination demonstrated the most effective biofilm-eradicating effect against the 48-h pre-formed biofilms of *K. pneumoniae* (**Figure 5A**). The results of live bacterial counting (**Figure 5B**) showed that, in contrast to the control, the number of live bacteria in the biofilms was significantly reduced after treatment with triple combinations of SPR741, CLA, and E. For example, the combination of 8 μg/mL SPR741 with 16 μg/mL E showed a moderate effect against ATCC 4352 biofilms among the single and dual combinations, resulting in a 3.33 Log10 CFU/mL reduction in bacterial numbers, when 16 µg/mL CLA was added, presented the best killing effect, reducing the number of bacteria by 4.21 Log10 CFU/mL. For the KPLUO strain, the combination of 8 µg/mL SPR741 with 16 µg/mL CLA and 16 µg/mL E presented the best bactericidal activity, reducing the number of bacteria in the biofilm by 3.0 Log10 CFU/mL. Similarly, the CLSM images also showed that SPR741 combined with CLA and E exerted significant biofilm eradication activities, as compared to the control or single and dual combinations (**Figure 5C**), with the green fluorescence signal intensity of viable bacteria markedly decreased and the density of biofilm significantly weakened.

### Synergistic *In Vivo* Bactericidal Activity of the Triple Antibiotic Combination of SPR741, E, and CLA

**Figure 6:**
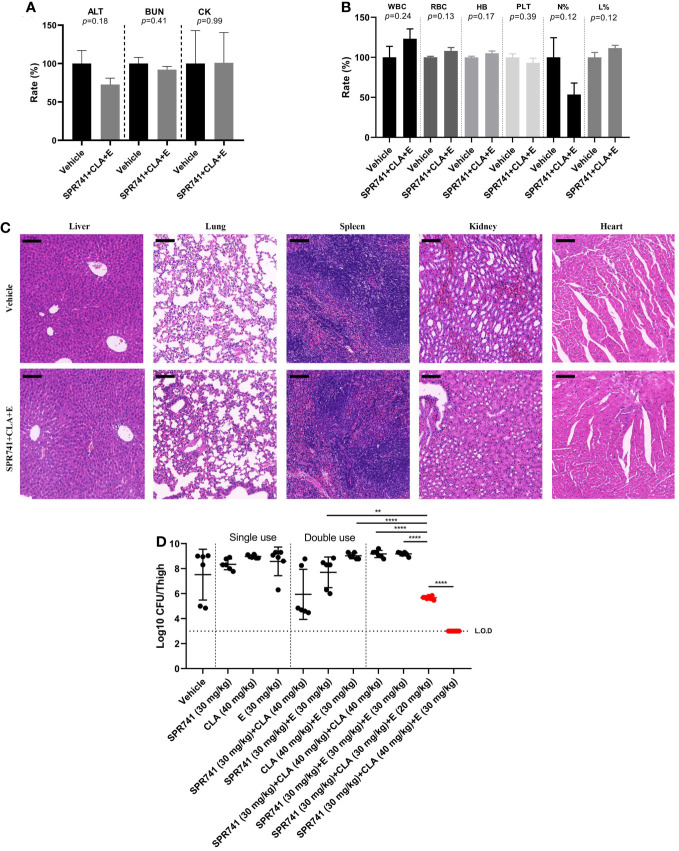
SPR741 combined with CLA and E displayed efficacy in a neutropenic murine thigh infection model, without detectable obvious toxicity. Biochemical routine examination **(A)**, blood cell analysis **(B)**, and hematoxylin-eosin staining **(C)** (from left to right: liver, lung, spleen, kidney and heart tissues. Scale bar: 100 µm) after subcutaneously administrating 30 mg/kg SPR741, 30 mg/kg E, and 40 mg/kg CLA in ICR mice. Saline with 1% DMSO was used as control. **(D) **As compared to mono or double antibiotics, SPR741 had potent synergy in triple combination with CLA and E, on reducing the bacterial load in PDR KP LH2020-related murine thigh infection model. Horizontal dotted line indicates the detection limit (LO.D). And the mono/double/triple dose treatment was separated using a vertical dotted line. The red dots indicate the triple treatment group of SPR741+CLA+E, *p<0.05; **p<0.01; ***p<0.001; ****p<0.0001.

Before *in vivo* efficacy testing, the *in vivo* toxicity was evaluated by subcutaneous administration of 30 mg/kg SPR741, 30 mg/kg E, and 40 mg/kg CLA into ICR mice. In the routine biochemical examination, there was no statistical difference among the various parameters including ALT, BUN, and CK (*P*>0.05) (**Figure 6A**). The blood cell analysis results showed that there was no statistical difference among the various parameters, including white blood cell count, red blood cell count, hemoglobin quantification, platelet count, neutrophil classification (N%), and lymphocyte classification (L%) (**Figure 6B**). Furthermore, histopathological analysis showed that compared with the control group, the triple antibiotic combination group did not cause significant injury to the tissues, and no granulocyte infiltration, hemorrhage phenomenon, or morphological changes were observed (**Figure 6C**). These results suggested that no obvious toxicity was found after treatment with SPR, CLA, and E, indicating that these triple antibiotics are relatively safe to be used together.

A neutropenic murine thigh infection model was used to evaluate therapeutic efficacy *in vivo.* Results showed that the triple combination of 30mg/kg SPR741, 30 mg/kg E, and 40 mg/kg CLA had the most significant anti-microbial effect, as demonstrated by the reduced bacterial load of 4.52 Log10 CFU/thigh compared to vehicle group, which was below the detection limit (**Figure 6D**). Even lowering the administration doses of CLA and E in the triple-drug combination to 30 mg/kg and 20 mg/kg, respectively, it is still demonstrated excellent *in vivo* efficacy, resulting in a reduction in bacterial load of 1.83 Log10 CFU/thigh. It was better than the double-drug combination of 30 mg/kg SPR741 + 40 mg/kg CLA, which reduced the bacterial count by 1.58 Log10 CFU/thigh. Moreover, the anti-bacterial efficacy of the triple combination of 30 mg/kg SPR741 + 30mg/kg E + 40mg/kg CLA was more effective than those of the 30 mg/kg SPR741 + 30 mg/kg E + 30 mg/kg E and 30mg/kg SPR741 + 40mg/kg CLA + 40mg/kg CLA combinations. This indicates significant synergistic anti-microbial efficacy of SPR741+E+CLA, rather than additional effects of SPR741 and macrolide antibiotics *in vivo*. It could be seen that only when SPR741 was used in combination with CLA and E (triple combination) could it demonstrate remarkable bactericidal activity *in vivo.*

## Discussion

In this study, we innovatively applied SPR741 and macrolide antibiotics in a triple drug combination against XDR and PDR resistant *K. pneumoniae*. To our knowledge, no study has reported the triple antibiotic combination use of SPR741 with macrolides, besides, this is the first study to report the effects of SPR741 against *K. pneumoniae* biofilms and persister cells. Our study found that SPR741 combined with macrolide antibiotics exhibited strong anti-microbial effects against *K. pneumoniae*, killed persister cells, and eradicated well-established biofilms. These results indicated that SPR741 combined with macrolides is a promising therapeutic candidate for XDR and PDR *K. pneumoniae* infections.

The PB analogue SPR741 lacks significant direct anti-microbial activity against Gram-negative bacteria; however, when combined with partner antibiotics that have limited or no anti-bacterial activity against Gram-negative species, SPR741 expands the spectrum of the partner antibiotic, and displays the potential to reduce the MIC values and achieve better anti-microbial efficacy (Mendes et al., 2020b). Moreover, SPR741 has been shown to be well tolerated in both single and multiple dose studies, and most of the drug-related treatment-emergent adverse events were mild (Eckburg et al., 2019). In addition, compared with PB, the safety profile of SPR741 was significantly improved, and its non-clinical nephrotoxicity was significantly reduced (Corbett et al., 2017). Antibiotic susceptibility testing, checkerboard assay, and viable cell counting (Lenhard et al., 2017; Pryjma et al., 2018; Aye et al., 2020) are the most commonly used approaches to evaluate the combined anti-bacterial effect of triple antibiotic combinations. Similarly, in this study, we used antibiotic susceptibility test, checkerboard combination assay, and bacterial counting method, to validate the synergistic anti-microbial effects of SPR741 combined with macrolide antibiotics, from various aspects.

QS is a cell-to-cell communication system that can regulate the process of biofilm formation, survival genes, and expression of various virulence factors (Li and Zhao, 2020; Seleem et al., 2020). Blocking the QS signal system can attenuate bacterial pathogenicity and slow the emergence of microbial resistance (Suga and Smith, 2003). Thus, the development of novel QS inhibitors (QSIs) is of great value for clinical use. The currently reported strategies for exploiting QSIs include synthesis of known QSI derivatives, modification of existing quorum-quenching enzymes, search for QSIs in natural products, and identification of approved drugs as QSIs (Zhou et al., 2020b). Our research used macrolide antibiotics as QSIs, which can shorten the development cycle of QS inhibiting agents from discovery to clinical application. Furthermore, some studies reported that CLA could induces the release of LL-37-bearing neutrophil extracellular traps, which are able to inhibit *Acinetobacter baumannii* growth and biofilm formation in an LL-37-dependent manner (Konstantinidis et al., 2016; Arampatzioglou et al., 2018). Hence, we speculate that there may have some other mechanisms between CLA and E that contributes to their synergistic antimicrobial action, and future studies still needed to elucidate the synergistic effects between these three drugs.

Our results also showed that for different *K. pneumoniae* strains, the combination of SPR741 with macrolides demonstrated varied bactericidal potency against *K. pneumoniae* and its persister cells. We speculated that the destruction of the bacterial outer membrane by SPR741 could facilitate macrolides crossing through the damaged outer membrane and enter into bacterial interior to exert antibacterial activity, thereby killing the *K. pneumoniae* cells. However, different cell wall compositions, resistance mechanism and genetic backgrounds of the clinical isolates may lead to different susceptibilities to macrolides even in the presence of SPR741. However, the exact mechanisms are still needed to be further investigated. In general, in our study, drug combinations showed the effective bactericidal activity against *K. pneumoniae* planktonic bacteria and persister cells. However, according to the time-killing curves, there was a rapid regrowth for some strains after 6-8 h treatment. Several studies (Yau et al., 2009; Skarp et al., 2019) have reported this phenomenon, which may be related to antibiotic degradation, inoculation effects and selection for existing or emerging resistant subpopulations (Huang et al., 2017). But this problem could be solved by optimizing the administration, because generally in clinical antibiotic use, the antibiotics could be maintained at the effective concentration by increasing the frequency of administration according to its PD/PK parameters, which can effectively kill the bacteria and prevent the development of drug resistance.

Some studies have analyzed the pharmacokinetic properties of SPR741 in detail (Eckburg et al., 2019), and their results suggested that the average plasma concentration of SPR741 reached its peak approximately 1 h after a single intravenous injection and decreased over 24 h. SPR741 showed a linear and proportional pharmacokinetic curve when administered as a single 1 h intravenous infusion of 100–800 mg, with a mean half-life (t1/2) ranging from 2.0 to 3.8 h. When SPR741 was administered in multiple doses, the apparent terminal elimination half-life was 2.2 h on day 1 and 14h on day 14, while the t1/2 of E or CLA after intravenous administration to rats were 1.8 and 3.3 h, respectively. Eight hours after dosing, the concentration of the drug in the tissues quickly reaches its maximum value, and is mainly distributed in organs and peripheral tissues, including the liver, lungs, kidneys, and skin (Kobuchi et al., 2020). Furthermore, research has shown that simultaneous intravenous injection of 400 mg SPR741 and any partner antibiotics (ceftazidime, aztreonam, and piperacillin-tazobactam) had no significant effect on the concentration-time profile of SPR741 and partner antibiotics. Another study showed that co-administration of AZM (a 15-membered macrolide) and SPR741 did not alter the pharmacokinetic parameters of SPR741 (Stainton et al., 2018), implying that SPR741 combined with macrolide antibiotics may not change the respective pharmacokinetic profiles.

In conclusion, SPR741 in combination with macrolide antibiotics showed strong synergistic anti-microbial, anti-persistent, and anti-biofilm activity against XDR and PDR *K. pneumoniae*. Moreover, it showed excellent anti-bacterial efficacy *in vivo*. These findings indicated that SPR741-macrolides triple combinations are promising therapeutic candidates for *K. pneumoniae* infections.

## Data Availability Statement

The original contributions presented in the study are included in the article/**Supplementary Material**. Further inquiries can be directed to the corresponding authors.

## Ethics Statement

All animal procedures were performed in accordance with the Ethics Committee of the Third Xiangya Hospital, Central South University (no: 2019sydw0233).

## Author Contributions

PS, YQL, and YW conceived and designed the experiments. PS and YQL performed most of the experiments and composed the manuscript. PS analyzed and plotted the results. LX, YML, ZL, SL, and ZH provided some methods needed for this research. YW supervised the entire study. All authors contributed to the article and approved the submitted version.

## Funding

This study was supported by the National Natural Science Foundation of China (No: 82072350) and the Natural Science Foundation of Hunan Province (No: 2021JJ40944).

## Conflict of Interest

The authors declare that the research was conducted in the absence of any commercial or financial relationships that could be construed as a potential conflict of interest.

## Publisher’s Note

All claims expressed in this article are solely those of the authors and do not necessarily represent those of their affiliated organizations, or those of the publisher, the editors and the reviewers. Any product that may be evaluated in this article, or claim that may be made by its manufacturer, is not guaranteed or endorsed by the publisher.
