# Supplementary material for: SPR741, Double- or Triple-Combined With Erythromycin and Clarithromycin, Combats Drug-Resistant Klebsiella pneumoniae, Its Biofilms, and Persister Cells
Source: Front Cell Infect Microbiol. 2022 Mar 18;12:858606. doi: 10.3389/fcimb.2022.858606 (PMC8971605; doi:10.3389/fcimb.2022.858606)
Supplement: Supplementary file 1 [file DataSheet_1.docx]

**Supplementary Material**

**1 Table S1.** Double or triple antibiotics combination between SPR741, CLA and E against PDR *K. pneumoniae* LH2020.

| Strain | Drugs | E | MIC_singly_ | MIC_in combination_ | MIC_singly_/ MIC_in combination_ | FICI | Outcome |
| --- | --- | --- | --- | --- | --- | --- | --- |
| LH2020 | SPR741  CLA | \ | 128  256 | 32  32 | 0.25  0.125 | 0.375 | Synergy |
|  | SPR741  E | \ | 128  256 | 2  64 | 0.0156  0.25 | 0.266 | Synergy |
|  | SPR741  CLA | 8 | 128  256 | 8  32 | 0.0625  0.125 | 0.187 | Synergy |
|  | SPR741  CLA | 16 | 128  256 | 8  16 | 0.0625  0.0625 | 0.125 | Synergy |
|  | SPR741  CLA | 32 | 128  256 | 8  16 | 0.0625  0.0625 | 0.125 | Synergy |

**Table S2.** Double or triple antibiotics combination between SPR741, CLA and E against XDR *K. pneumoniae* KPLUO, KPWANG and type strain ATCC 700603.

| Strains | Drugs | E | MIC_singly_ | MIC_in combination_ | MIC_singly_/ MIC_in combination_ | FICI | Outcome |
| --- | --- | --- | --- | --- | --- | --- | --- |
| KPLUO | SPR741  CLA | \ | 64  128 | 4  16 | 0.0625  0.125 | 0.187 | Synergy |
|  | SPR741  E | \ | 128  512 | 16  16 | 0.125  0.0312 | 0.156 | Synergy |
|  | SPR741  CLA | 8 | 64  128 | 2  2 | 0.0312  0.0156 | 0.0468 | Synergy |
| KPWANG | SPR741  CLA | \ | 64  128 | 2  4 | 0.03125  0.03125 | 0.0625 | Synergy |
|  | SPR741  E | \ | 256  512 | 8  16 | 0.03125  0.03125 | 0.0625 | Synergy |
|  | SPR741  CLA | 4 | 64  128 | 2  2 | 0.03125  0.0156 | 0.0468 | Synergy |
| ATCC 700603 | SPR741  CLA | \ | 128  128 | 8  2 | 0.0625  0.0156 | 0.0781 | Synergy |
|  | SPR741  E | \ | 128  512 | 16  32 | 0.125  0.0625 | 0.1875 | Synergy |
|  | SPR741  CLA | 4 | 128  128 | 4  2 | 0.03125  0.0156 | 0.0468 | Synergy |

1. **Supplementary Figures**

**
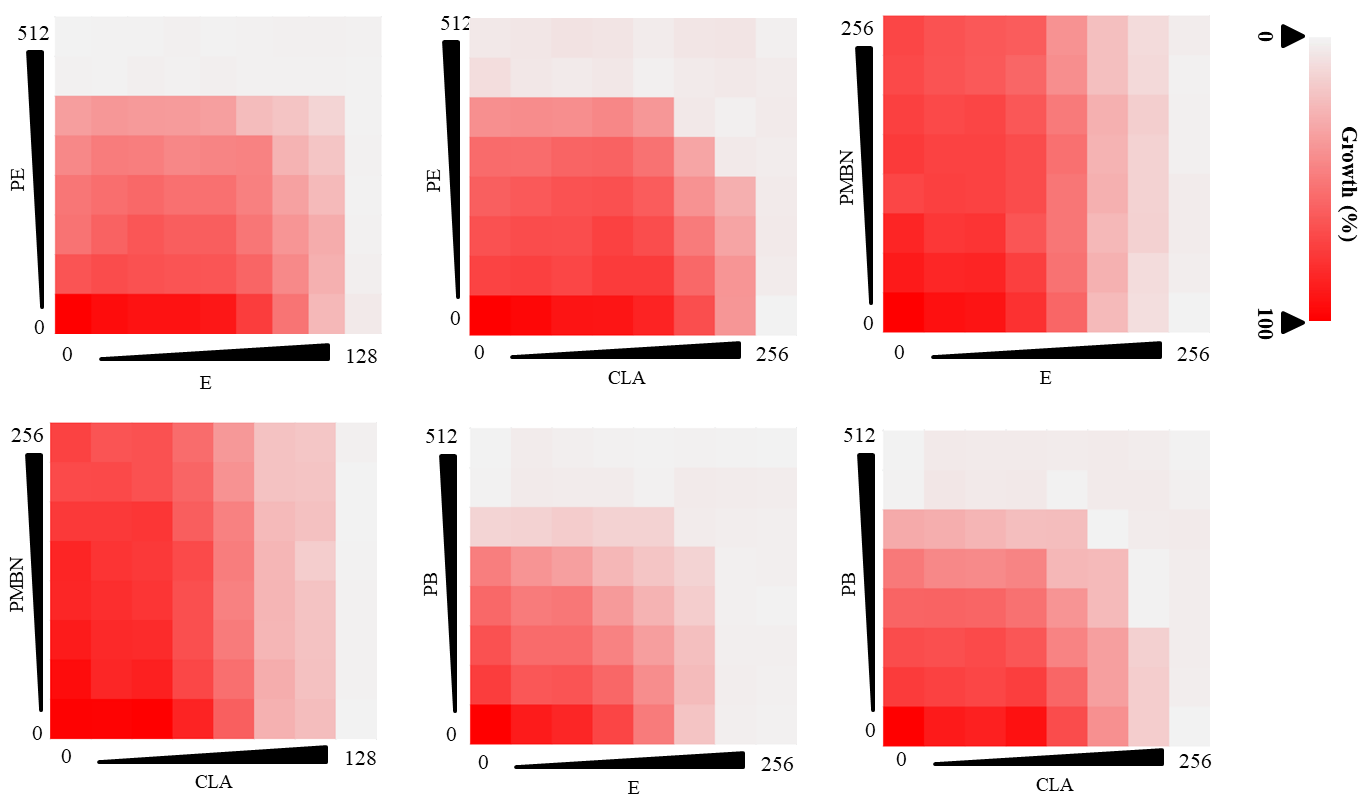
**

**Figure S1.** Antibacterial effects of polymyxin E (PE), polymyxin B nonapeptide (PMBN), and polymyxin B (PB) combined with erythromycin (E) or clarithromycin (CLA) against LH2020 were determined using checkerboard assay.

**
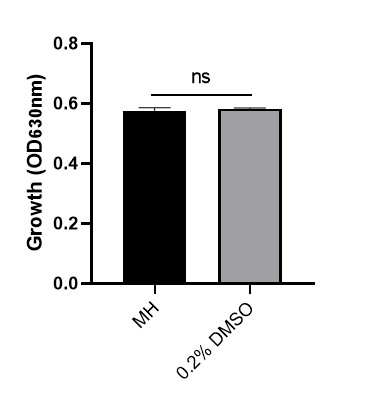
**

**Figure S2**. Growth of *K. pneumoniae* LH2020 in MH broth with or without the addition of DMSO. There was no statistical difference in OD_630nm_ values between MH broth with or without 0.2% DMSO.


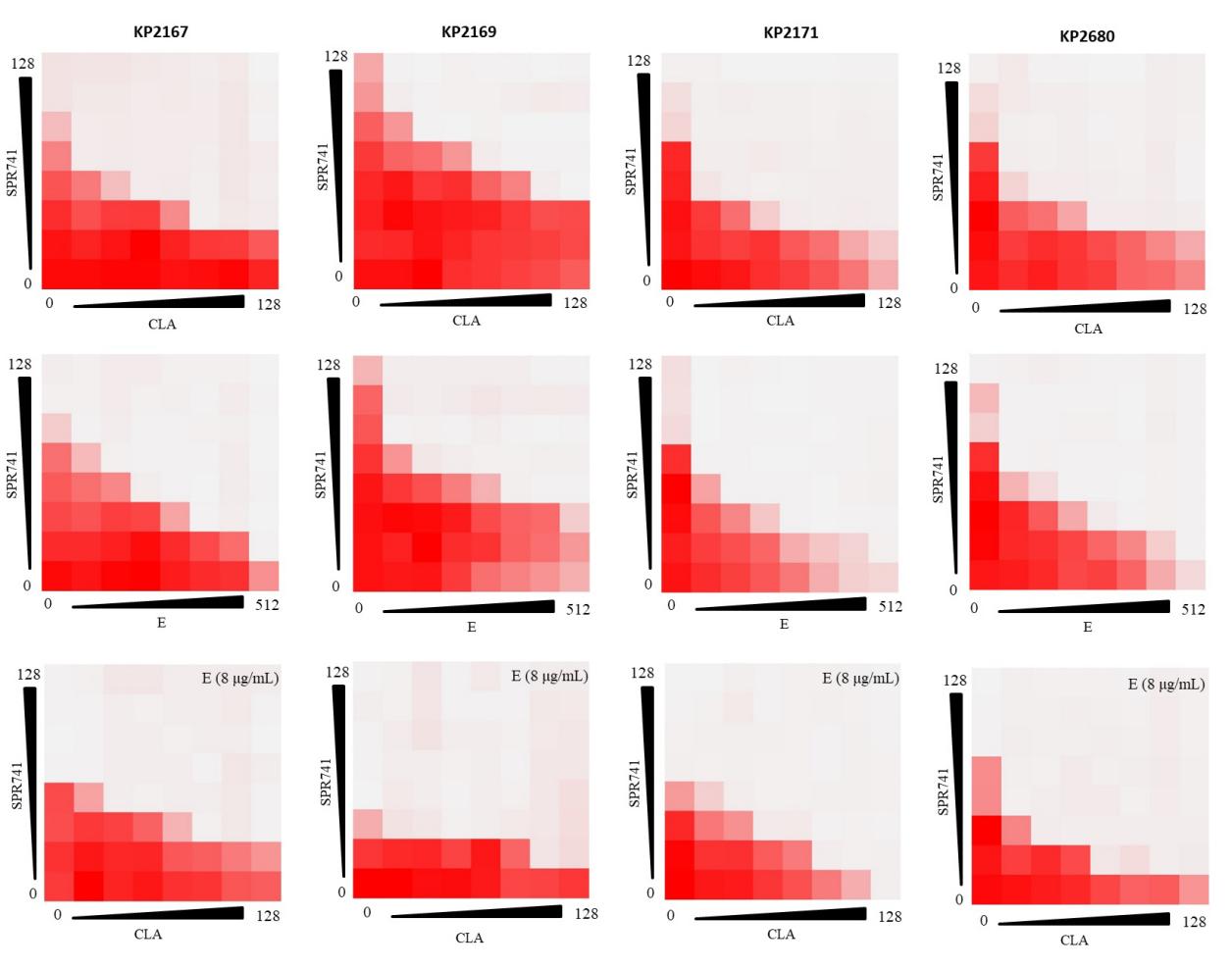


**Figure S3.** Combination anti-microbial activities of SPR741, CLA, and E against MDR strain of *K. pneumoniae*. Double antibiotic combination of SPR741 with CLA(A) or E (B), and triple combination of SPR741 with CLA and 8 µg/mL of E (C) against MDR *K. pneumoniae* KP2167, KP2169, KP2171and KP2680 were determined using checkerboard assay.
